# Calcitonin gene-related peptide (CGRP) and its receptor components in human and rat spinal trigeminal nucleus and spinal cord at C1-level

**DOI:** 10.1186/1471-2202-12-112

**Published:** 2011-11-10

**Authors:** Sajedeh Eftekhari, Lars Edvinsson

**Affiliations:** 1 Department of Clinical Sciences, Division of Experimental Vascular Research, Lund University, Lund, Sweden

## Abstract

**Background:**

Calcitonin gene-related peptide (CGRP) has a key role in migraine pathophysiology and is associated with activation of the trigeminovascular system. The trigeminal ganglion, storing CGRP and its receptor components, projects peripheral to the intracranial vasculature and central to regions in the brainstem with Aδ- and C-fibers; this constitutes an essential part of the pain pathways activated in migraine attacks. Therefore it is of importance to identify the regions within the brainstem that processes nociceptive information from the trigeminovascular system, such as the spinal trigeminal nucleus (STN) and the C1-level of the spinal cord. Immunohistochemistry was used to study the distribution and relation between CGRP and its receptor components - calcitonin receptor-like receptor (CLR) and receptor activity modifying protein 1 (RAMP1) - in human and rat STN and at the C1-level, using a set of newly well characterized antibodies. In addition, double-stainings with CGRP and myelin basic protein (MBP, myelin), synaptophysin (synaptic vesicles) or IB4 (C-fibers in general) were performed.

**Results:**

In the STN, the highest density of CGRP immunoreactive fibers were found in a network around fiber bundles in the superficial laminae. CLR and RAMP1 expression were predominately found in fibers in the spinal trigeminal tract region, with some fibers spanning into the superficial laminae. Co-localization between CGRP and its receptor components was not noted. In C1, CGRP was expressed in fibers of laminae I and II. The CGRP staining was similar in rat, except for CGRP positive neurons that were found close to the central canal. In C1, the receptor components were detected in laminae I and II, however these fibers were distinct from fibers expressing CGRP as verified by confocal microscopy.

**Conclusions:**

This study demonstrates the detailed expression of CGRP and its receptor components within STN in the brainstem and in the spinal cord at C1-level, and shows the possibility of CGRP acting postjunctionally in these areas putatively involved in primary headaches.

## Background

Migraine is considered as a neurovascular disorder affecting more than 10% of the general population. Calcitonin gene-related peptide (CGRP) has a key role in migraine, where levels of CGRP are increased during acute migraine attacks [1]. CGRP is expressed throughout the central and peripheral nervous systems, consistent with control of vasodilatation and transmission of nociceptive information. In migraine, CGRP is released from the trigeminal vascular system. At peripheral synapses, CGRP results in vasodilatation via receptors on the smooth muscle cells. At central synapses, CGRP has been suggested to act postjunctionally on second-order neurons to transmit pain centrally via the brainstem and midbrain to higher cortical pain regions [2].

There are two forms of this peptide; (i) αCGRP, which is predominantly expressed in the nervous system, and (ii) βCGRP, which is primarily expressed in the enteric sensory system. In the central nervous system (CNS), CGRP is expressed in several regions such as the striatum, amygdalae, hypothalamus, colliculi, brainstem, cerebellum and the trigeminal complex [3-7]. Moreover, CGRP is found in primary spinal afferent C- and Aδ-fibers, which project to the brainstem. However, CGRP and its receptor components have not fully been studied in man due to the fact that the receptor components only fairly recently were characterized.

The receptor for CGRP consists of a complex of a seven transmembrane-spanning protein, calcitonin receptor-like receptor (CLR), a single transmembrane-spanning protein designated receptor activity modifying protein 1 (RAMP1) [8] and an intracellular protein, receptor component protein (RCP) [9]. Recently, CGRP antagonists have been developed with clinical efficacy for the treatment of acute migraine attacks [10-12]. Consequently, it is of considerable importance to clarify where the CGRP receptor is expressed which would indicate possible sites for the therapeutic effect of these antagonists. Hence, studies have focused on mapping CGRP and its receptor components in the trigeminovascular system and in the brainstem as recently reviewed [13].

A migraine active region has been demonstrated in the brainstem with positron emission tomography (PET) [14-16]. It has been hypothesized that brainstem stimulation can cause activation of the trigeminovascular system, resulting in CGRP-dependent vasodilatation [17].

We have investigated in detail the distribution and relationship of CGRP and its receptor components within human and rat spinal trigeminal nucleus (STN) in the brainstem and in the spinal cord at C1-level, using a set of newly characterized antibodies for immunohistochemistry [7]. Our main findings in the present work were that CGRP and the receptor components appear in different structures/regions of STN, and in lamina I and II at C1-level, but do not co-localize. This suggests that C-fiber released CGRP acts postjunctionally on fibers expressing CLR/RAMP1 in these regions.

## Methods

### Postmortem human tissue samples

Samples of STN and C1 were obtained at autopsy from adult subjects in accordance with the Faculty of Medicine University of Szeged guidelines for ethics in human tissue experiments and were approved by the local Hungarian Ethics Committee. The tissue was bilaterally removed from 6 subjects (3 female; 3 male) with an age span of 65 to 86 years. None of the subjects suffered from any central nervous system disease and the cause of death was related to heart failure, septicemia or cancer. The tissues were collected within 24 to 36 hrs after death.

The samples were immersed overnight in fixative consisting of 4% paraformaldehyde (PFA) and in 0.1 mol/*l *phosphate buffer, pH 7.2. After fixation, the specimens were rinsed in sucrose-enriched (10%) Tyrode solution overnight, frozen and stored at -80°C. The samples were embedded in a gelatin medium (30% egg albumin and 3% gelatin in distilled water) and cryosectioned at 12 μm. The sections were stored at - 20°C until use.

### Rat tissue samples

Brainstems were quickly removed from 5 male Sprague-Dawley rats weighing 300-350 g (Scanbur, Stockholm, Sweden). The STN samples were dissected at bregma -14.08, corresponding the caudal part of STN, Sp5C (using brain atlas Paxinos and Watson, second edition, 1986) and C1 were dissected at C1 vertebra. The tissues were immediately placed in 4% PFA and fixed for 2-4 hrs. After fixation the tissues were rinsed in raising concentrations of sucrose in Sörensen's phosphate buffer, embedded, sectioned and stored as the human samples.

Brainstems from 2 additional rats were after removal kept in the refrigerator at +4°C for 24 hrs before they were treated as above (to mimic the autopsy situation in man).

Also, comparison was made to tissue obtained from 2 additional rats that were perfusion fixed with 4% PFA (data not shown). We found no difference in the immunostaining patterns between the two procedures. The experiments were approved by the University Animal Ethics Committee (M8-09), Lund University, Sweden.

### Hematoxylin-Eosin staining

Human and rat sections were stained with Hematoxylin-Eosin (Htx-Eosin) using a standard protocol (Htx 3 min, water rinse, Eosin 1 min) for orientation and examination of the tissue condition. The areas within the rat and human brainstem were identified by the use of a brain atlas (Paxinos and Watson, second edition, 1986, and Koutcherov et al. chapter 10 in The Human Nervous System). Sections with STN (the caudal subdivision) or C1, were used and adjacent sections were employed for immunohistochemistry.

### Immunohistochemistry

Sections were thawed and washed for 10 min in PBS pH 7.2 containing 0.25% Triton X-100 (PBST). The sections were blocked for 1 hr in blocking solution of PBS and 5% normal donkey or goat serum (depending on species origin of the secondary antibody). After blocking, the sections were incubated overnight at +4°C for single or double immunolabelling with primary antibodies against CGRP, CLR and RAMP1. Anti-human CLR (3152) and RAMP1 (844) were used for the human material, anti-rat CLR (3155, 132) and RAMP1 (3155) were used for the rat material. For detailed description of the primary antibodies, see Table 1.

**Table 1:** Details on primary antibodies used for immunohistochemistry

Name and product code	Host	Dilution	Detects	Supplier
Calcitonin receptor-like receptor (CLR) 3152	Rabbit	1:500	C-terminal of human CLR	Merck & Co., Inc, USA
CLR 3155	Rabbit	1:500	C-terminal of rat CLR	Merck & Co., Inc, USA
CLR 132	Sheep	1:100	C-terminal of rat CLR	Merck & Co., Inc, USA
Receptor activity-modifying protein 1 (RAMP1) 844	Goat	1:100	C-terminal of human RAMP1	Merck & Co., Inc, USA
RAMP1 3158	Rabbit	1:500	C-terminal of rat RAMP1	Merck & Co., Inc, USA
Calcitonin gene-related peptide (CGRP), polyclonal, B47-1	Rabbit	1:1600	rat CGRP	Europroxima; Arnhem, The Netherlands
CGRP, monoclonal, ab81887	Mouse	1:100	rat α-CGRP	Abcam; UK
Myelin basic protein (MBP) polyclonal, A0623	Rabbit	1:200	Myelin, Schwann cells	Dako; Cophenhagen, Denmark
Synaptophysin, polyclonal, A0010	Rabbit	1:100	Synaptic vesicle protein	Dako; Copenhagen, Denmark
Isolectin IB4 Alexa flour 594 conjugate, I21413		1:50	C-fibers (in general)	Invitrogen; CA, USA

The primary antibodies were diluted in PBST containing 1% BSA and 3% normal serum. After incubation with primary antibodies, sections were equilibrated to room temperature, rinsed in PBST for 3 × 15 min and exposed to secondary antibodies (for details, see Table 2) in PBST and 1% BSA for 1 hr at room temperature. The sections were subsequently washed with PBST for 3 × 15 min. Vectashield, an anti-fading medium, containing DAPI (Vectashield, Vector Laboratories., Burlingame CA) or glycerol in PBS were used as mounting media.

In addition, double immunostainings for CGRP together with either myelin basic protein (MBP), synaptic vesicle protein (synaptophysin) or Isolectin IB4 (IB4) were performed (Table 1). For all double immunostainings, the antibodies were applied separately and not mixed as a cocktail.

**Table 2:** Secondary antibodies used for immunohistochemistry

Conjugate and host	Against	Dilution	Supplier
FITC (goat)	anti-rabbit	1:100	Cayman Chemical, Ann Arbor, MI
Cy^2 ^(donkey)	anti-rabbit	1:200	Jackson Immuoresearch, West Grove, PA
Texas-Red (donkey)	anti-rabbit	1:200	Jackson Immuoresearch, West Grove, PA
Alexa 488 (donkey)	anti-goat	1:400	Invitrogen, CA
Cy^2 ^(donkey)	anti-goat	1:200	Jackson Immuoresearch, West Grove, PA
Cy^3^(donkey)	anti-goat	1:200	Jackson Immuoresearch, West Grove, PA
Texas-Red (donkey)	anti-mouse	1:200	Jackson Immuoresearch, West Grove, PA
Texas-Red (donkey)	anti-sheep	1:200	Jackson Immuoresearch, West Grove, PA

### Controls and DAB staining

Omission of the primary antibody served as negative controls for all antibodies.

To evaluate secondary antibody staining, three different secondary antibodies (Table 2) were tested together with CLR or RAMP1, respectively.

Preabsorption controls with blocking peptides (details on these have been described before in [7]) were performed with all CLR and RAMP1 primary antibodies. Concentrations of the antibodies were the same as described in Table 1, peptide concentrations were 100:1. The blocking peptides were resuspended in PBS and then incubated at +4°C overnight in PBST containing 1% BSA and 3% normal serum, with or without primary antibodies. The immunostaining protocol was the same as described above. Sections incubated with antibodies alone versus blocked antibodies were compared.

In order to evaluate the fluorescence technique staining, 3,3'-diaminobenzidine (DAB) substrate together with Vectastain ABC kit standard PK-6100 (Vector Laboratories) was performed. In brief, sections were rinsed in PBST followed by incubation with methanolic hydrogen peroxidase (3% H_2_O_2 _and 10% MetOH in PBS) to remove endogenous activity. After incubation for 1 h with blocking solution of PBS and 5% normal swine or rabbit serum, the sections were incubated with primary antibodies against CGRP, CLR and RAMP1, at +4°C overnight. At the second day, sections were incubated with biotin-conjugated secondary antibodies, anti-rabbit or anti-goat for 1,5 h (1:400, Dako, Glostrup, Denmark). Visualization was achieved through the ABC kit using DAB/H_2_O_2_. Omission of primary antibody served as negative controls.

### Microscopic analysis

Sections were examined and images were obtained using a light- and epifluorescence microscope (Nikon 80i, Tokyo, Japan) coupled to a Nikon DS-2MV camera. Adobe Photoshop CS3 (v.8.0, Adobe Systems, Mountain View, CA) was used to visualise co-labelling by superimposing the digital images and to adjust brightness and contrast. In addition, confocal microscopy was performed using Nikon confocal microscope (EZ-cl, Germany), where detailed localization and/or co-localization of immunoreactivity were recorded using the confocal microscope. All pertinent questions were addressed with examination with confocal microscope analysis (see figures). The confocal microscopy was carried out using 20× or 60× oil immersion lenses. Z (frame) stacks were acquired using different laser channels one by one before next z position was acquired. Laser channels used were 488 nm excitation (filter 515/30) and 543 nm excitation (filter 605/75). Image analyses were conducted using NIS basic research software (Nikon, Japan). Briefly, z stacks were 3-dimensionally examined for detailed localization and distribution of immunoreactivity, and for possible co-localization of the antibodies used. The opportunity to move around 3-dimensionally within the 12 μm thick section allows for scrutinizing the detailed localization of the immunoreactivity, but also for thorough evaluation of the immunoreactive structures.

## Results

### Histology

**Figure 1:**
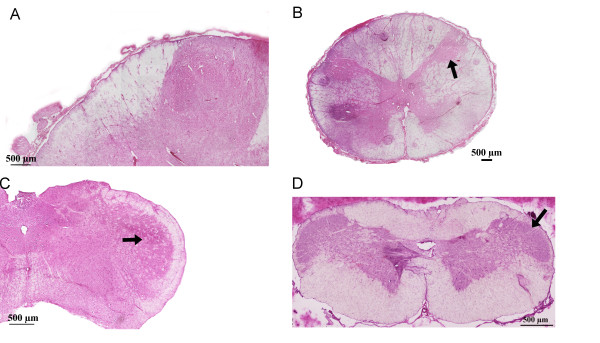
**Hematoxylin-Eosin staining of human and rat STN (caudal subdivision, Sp5C) and C1-level of the spinal cord**. (A) Human STN. (B) Human C1. Arrow points at the posterior horn. (C) Rat STN, (arrow). (D) Rat C1. Arrow points at laminae I and II.

Htx-Eosin stained sections from human STN (A), C1 (B) and rat STN (C) and C1 (D) are depicted in Figure 1. The human samples were considered qualitatively adequate for the immunofluorescence technique by and large comparable in structures to that found in rat.

### STN

#### CGRP immunoreactivity

**Figure 2:**
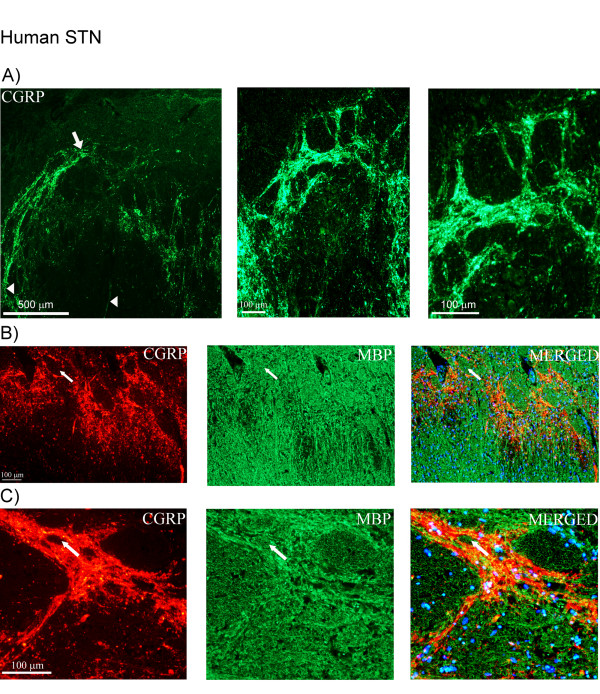
**CGRP and MBP (myelin marker) expression in human STN**. (A) Expression of CGRP in human STN. CGRP positive fibers are found in network around fiber bundles in the superficial laminae. Few fibers extend into the spinal trigeminal tract region (arrow) and the mid part of the brainstem (arrow heads). (B, C) Double-staining of CGRP (green) and MBP (red). Arrows point at MBP positive areas negative for CGRP. DAPI (blue), staining nuclei, is used in the merged pictures.

In human STN, the highest density of CGRP immunoreactive fibers was found in a network around fiber bundles in the superficial laminae (Figure 2A). In this area, few fibers extended into the spinal trigeminal tract region and into the mid part of the brainstem (Figure 2A). The CGRP immunoreactive fibers were thin and displayed "pearl-like" structures (Figure 2A). No CGRP positive cells were detected in the STN or in other regions within the brainstem at the examined level. Double-staining did not reveal any co-localization of CGRP and MBP (myelin marker) (Figure 2B,C).

**Figure 3:**
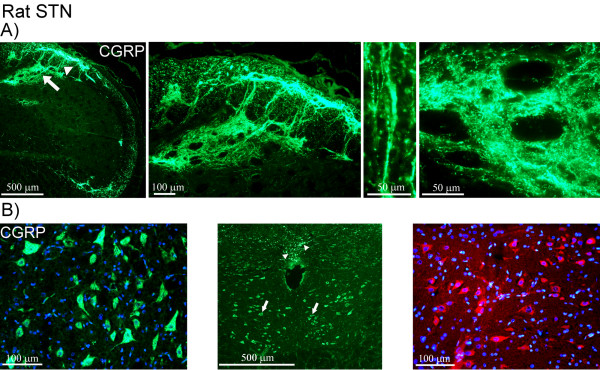
**CGRP expression in rat STN**. (A) CGRP staining of rat STN. Immunopositive fibers are mostly found in the superficial laminae (arrow) and some fibers extending into the spinal trigeminal tract region (arrow head). Higher magnification shows the staining pattern in detail around fiber bundles and displaying "pearl-like" immunoreactivity in the fibers. (B) CGRP positive neurons (visualized with two different primary antibodies; green and red) in the region of the inferior olive and hypoglossal nucleus (arrows). The staining displays granular-like pattern in the cytoplasm. CGRP positive fibers are also found close to the central canal (arrow heads).

**Figure 4:**
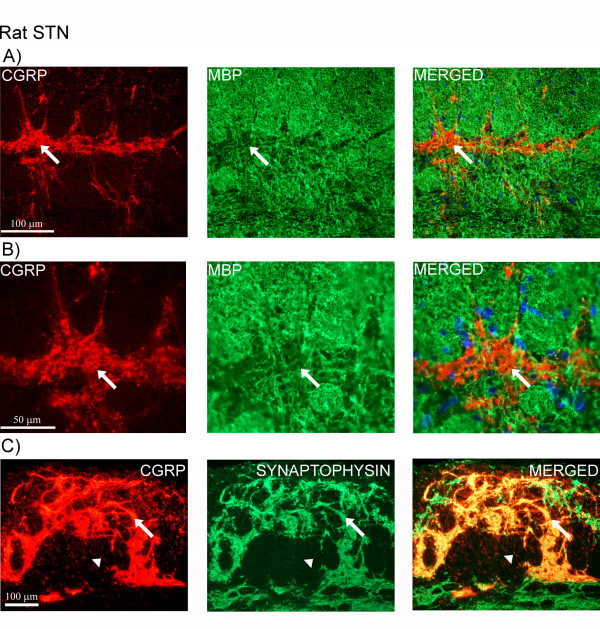
**Double-staining of CGRP and MBP or synaptophysin (synaptic vesicles)**. (A, B) Double-staining of CGRP (red) and MBP (green) in rat STN. Nuclei staining (DAPI, blue) is used in the merged pictures. Arrows point at CGRP positive areas negative for MBP. (C) CGRP (red) and synaptophysin (green) expression (arrows). Note that not all CGRP positive fibers co-localize with synaptophysin (arrow heads).

In rat STN, similar staining pattern of CGRP immunoreactivity was observed in the superficial laminae as in man, with some fibers extending into the spinal trigeminal tract (Figure 3A). The expression of CGRP was limited to fibers. However, in the region of the inferior olive and the hypoglossal nucleus, CGRP positive neurons were found. Here, CGRP immunoreactivity was revealed as intracellular, granular staining in the cytoplasm, visualized with the two different CGRP antibodies (Figure 3B). Double-staining did not reveal any co-localization of CGRP and MBP (Figure 4A,B). CGRP and synaptic vesicle protein (synaptophysin) double-staining showed co-expression of these two markers (Figure 4C). CGRP and synaptophysin revealed same results in human STN (data not shown).

#### CLR and RAMP1 immunoreactivity

**Figure 5:**
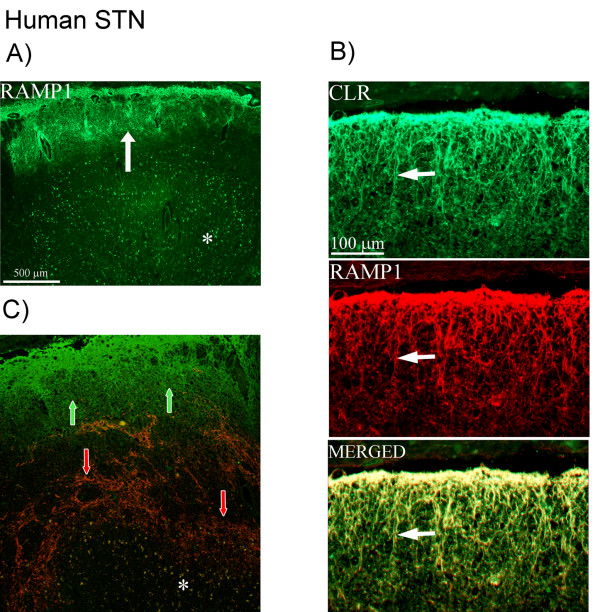
**CLR/RAMP1 and CGRP double-staining in human STN**. (A) Expression of RAMP1 in human STN. The expression is found in the spinal trigeminal tract region (arrow). (B) Co-expression of the receptor components, CLR (green) and RAMP1 (red), in fibers in the spinal trigeminal tract (arrows). (C) Confocal microscopy showing expression of CGRP (red, arrows) and RAMP1 (green, arrows). CGRP immunoreactivity is found in the superficial laminae, while the receptor component is expressed in the spinal trigeminal tract. No co-localization is found. Asterisks point at autofluorescent lipofuscin.

In human STN, the staining of the receptor components was not as distinct as the CGRP staining. Nevertheless, CLR and RAMP1 positive fibers were found in the spinal trigeminal tract region (Figure 5A). Double-staining of CLR and RAMP1 showed co-localization in the spinal trigeminal tract region (Figure 5B). No CLR or RAMP1 positive neurons were found. Double-staining did not reveal any co-localization of CGRP and RAMP1 (Figure 5C) or CGRP and CLR (data not shown).

**Figure 6:**
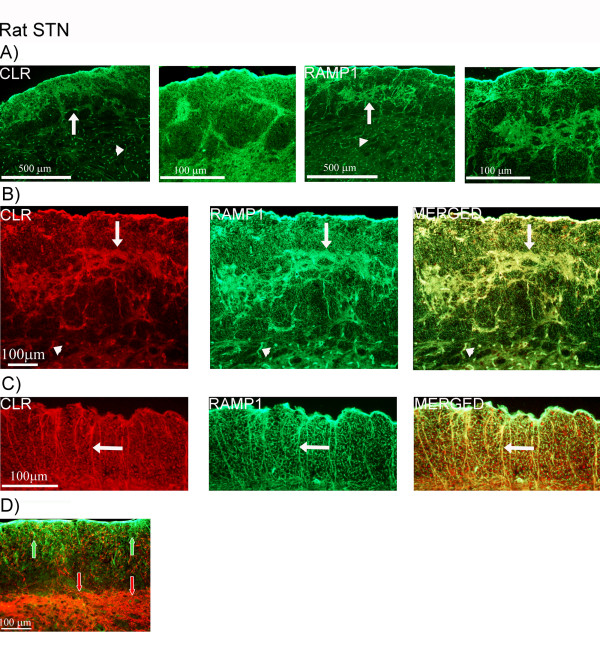
**CLR/RAMP1 and CGRP double-staining in rat STN**. (A) Expression of CLR and RAMP1 in rat STN. The receptor components are found around fiber bundles in the spinal trigeminal tract (arrows) and in the walls of capilliaries (arrow heads). (B, C) Double-staining of CLR (red) and RAMP1 (green). They co-localize in fiber bundles and in fibers spanning from the spinal trigeminal tract (arrows). Arrow heads point at co-expression in the capillaries. (D) CGRP and CLR double-staining, CGRP (red arrows) is mostly expressed in the superficial laminae while CLR (green arrows) is expressed in the spinal trigeminal tract. No co-localization is found.

Similar fiber staining pattern was seen in rat STN, however, the staining was more intense. In contrast to that of the human STN, positive CLR and RAMP1 staining was observed around fiber bundles within the spinal trigeminal tract (Figure 6A). Double-staining of CLR and RAMP1 showed co-localization in the spinal trigeminal tract region, in both fiber bundles (Figure 6B) and fibers spanning from the spinal trigeminal tract (Figure 6C). Double-staining of CGRP and the receptor components showed that CGRP is not expressed in the same fibers as CLR (Figure 6D) or RAMP1 (data not shown). No CLR or RAMP1 positive neurons were found. Interestingly, the receptor components were detected in the walls of capillaries within the rat brainstem (Figure 6).

### C1

#### CGRP immunoreactivity

**Figure 7:**
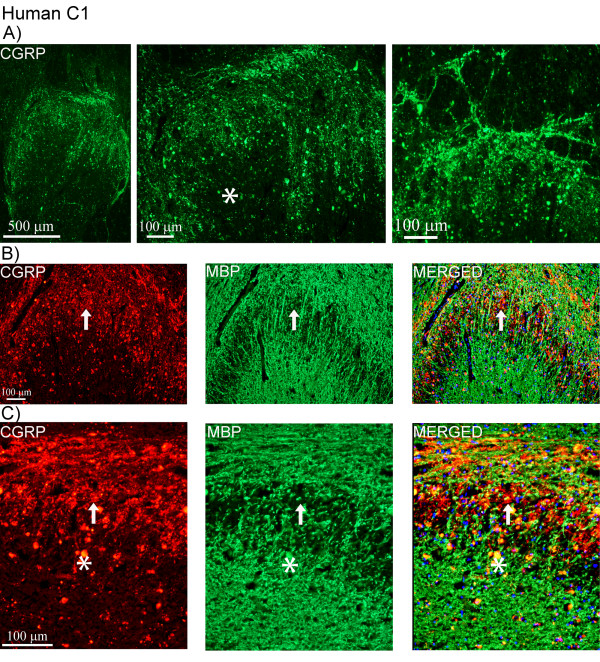
**CGRP and MBP expression in human C1**. (A) Expression of CGRP in human C1. Positive fibers are found within the posterior horn, mostly in laminae I and II. (B, C) Double-staining of CGRP (red) and MBP (green). Arrows point at CGRP positive areas negative for MBP. Nuclei staining (DAPI, blue) is used in the merged pictures. Asterisks point at autofluorescent lipofuscin, which appears yellow in the merged pictures.

At the C1-level of the human spinal cord, CGRP positive fibers were detected within the posterior horn of the gray matter, notably in laminae I and II (Figure 7A). In addition, few fibers reaching the deeper laminae were detected. No CGRP immunoreactive neurons were found in the human C1.

**Figure 8:**
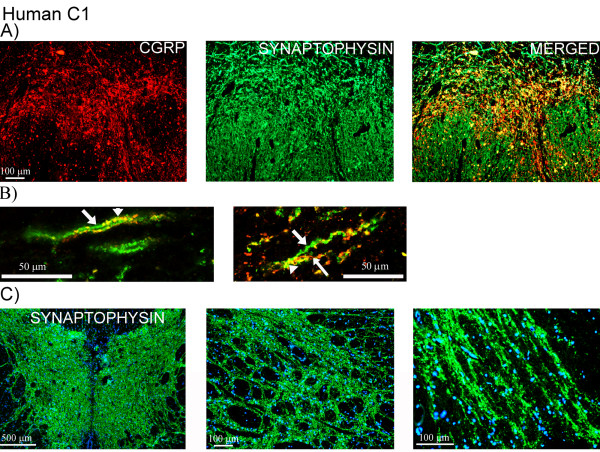
**CGRP and synaptophysin expression in human C1**. (A) Double-staining of CGRP (red) and synaptophysin (green) in human C1. Co-localization is shown in the merged picture. (B) Detailed confocal image showing staining of CGRP (red, thin arrow) and synaptophysin (green, thick arrow). Co-localization (yellow, arrow heads) is demonstrated in the fibers. (C) Synaptophysin and DAPI staining. "Pearl-like" synaptophysin immunoreactivity (green) is detected in all laminae in the fibers within C1-level.

Double-staining revealed no co-localization of CGRP and MBP, as the expression of the two markers was observed in different structures at the C1-level (Figure 7B,C). CGRP was detected in areas that were absent from MBP expression. Furthermore, CGRP and synaptophysin were expressed in the same laminae and structures (Figure 8A), which was confirmed by confocal microscopy (Figure 8B). Not all synaptophysin positive fibers expressed CGRP. Expression of synaptophysin was detected in all laminae within C1-level (Figure 8C).

**Figure 9:**
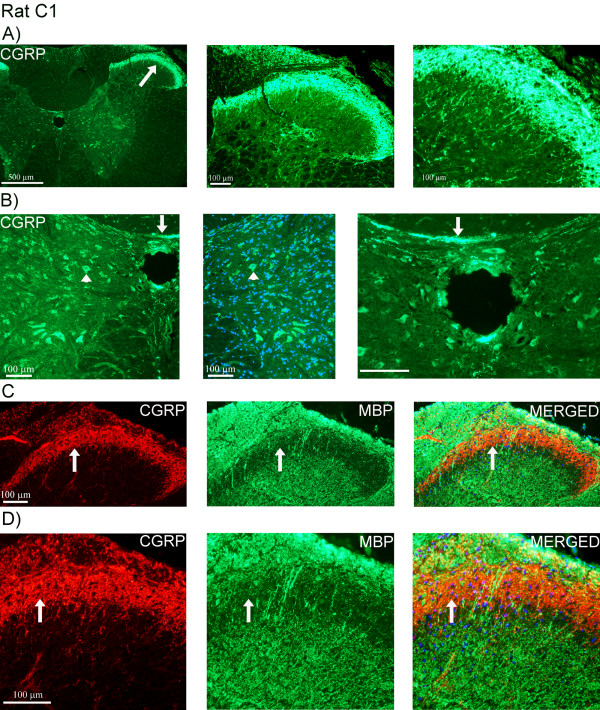
**CGRP and MBP expression in rat C1**. (A) CGRP expression in laminae I and II (arrow) in rat C1. (B) Transverse CGRP positive fibers (arrows) and CGRP positive neurons (arrow heads) are found close to the central canal. (C, D) Double-staining of CGRP (red) and MBP (green). Arrows point at CGRP positive areas negative for MBP. Nuclei staining (DAPI, blue) is used in the merged pictures.

**Figure 10:**
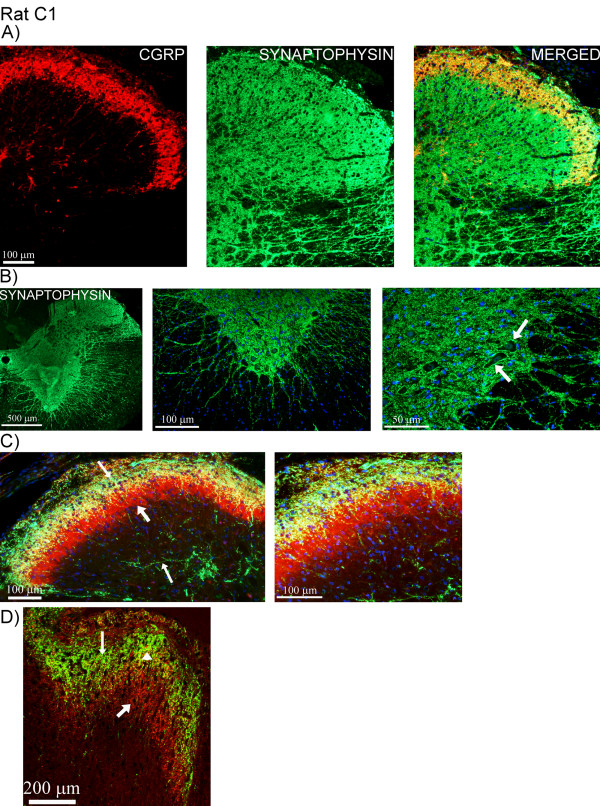
**Double-staining of CGRP and synaptophysin or IB4**. (A) Double-staining of CGRP (red) and synaptophysin (green) in rat C1. (B) Synaptophysin immunoreactivity is found all laminae, with fibers extending into to the white matter. Circular immunoreactive fiber formations are seen around neurons (arrows). (C) Double-staining of CGRP (green, thin arrows) and IB4 (red, thick arrow). IB4 is mostly expressed in laminae II/III. IB4 is also expressed in laminae I/II which could be seen as co-localization with CGRP. However, confocal microscopy (D) revealed that CGRP (green, thin arrow) and IB4 (red, thick arrow) are most often not expressed in the same fibers. Occasionally the two markers co-localize in fibers (arrow head).

The CGRP staining was similar in rat C1 compared to man (Figure 9A). In rat, transverse positive CGRP fibers were detected close to the central canal (Figure 9B). In addition, CGRP positive neurons were found close to the central canal (Figure 9B). Double-staining with CGRP and MBP (Figure 9C,D), CGRP and synaptophysin (Figure 10A) revealed similar results as those seen in the human samples. Synaptophysin expression was detected in all laminae, including circular immunoreactive fiber formations around neurons (Figure 10B). Double-staining with CGRP and the IB4 marker revealed CGRP and IB4 immunoreactivity in laminae I and II (Figure 10C). IB4 staining was brighter in laminae II/III, compared to laminae I and II. Confocal microscopy disclosed that CGRP and IB4 were most often not expressed in the same fibers. However, occasionally the slender CGRP positive fibers co-localized with the more stubby IB4 immunoreactive fibers (Figure 10D, additional file 1, movie).

#### CLR and RAMP1 immunoreactivity

**Figure 11:**
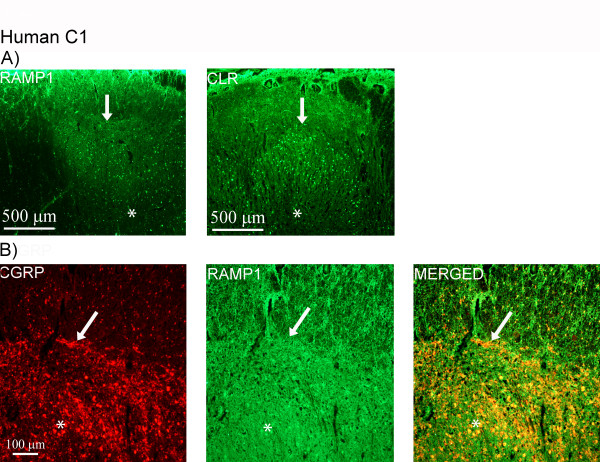
**CLR/RAMP1 and CGRP double-staining in human C1**. (A) Expression of RAMP1 and CLR in human C1. The receptor components are found within laminae I and II. (B) Double-staining of CGRP (red) and RAMP1 (green) shows that CGRP positive fibers (arrows) within laminae I and II do not co-localize with RAMP1. Asterisks point at autofluorescent lipofuscin.

In human C1, the receptor components were detected in laminae I and II, although the staining was weaker compared to the CGRP staining (Figure 11A). No positive cells for CLR or RAMP1 were found. Double-staining of CGRP and RAMP1 showed no co-localization (Figure 11B). Same results were observed with CGRP and CLR (data not shown).

**Figure 12:**
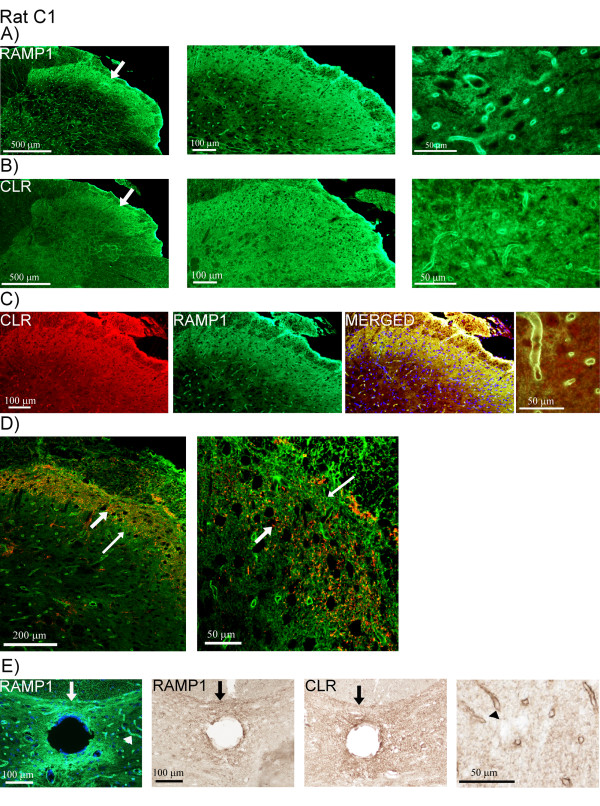
**CLR/RAMP1 and CGRP double-staining in rat C1**. (A) RAMP1 and (B) CLR expression in rat C1. The receptor components are found from Lissauer's tract and within laminae I and II (arrows). Higher magnification discloses expression in the walls of the capillaries. (C) Co-expression of CLR (red) and RAMP1 (green) in the laminae and the capillaries. (D) Double-staining of CGRP (red, thick arrows) and RAMP1 (green, thin arrows) using confocal microscopy. CGRP and RAMP1 are not co-expressed. (E) Fluorescence and DAB staining of the receptor components. Positive fibers are displayed close to the central canal (arrows), where also positive capillaries are found (arrow heads).

In rat C1, the staining for CLR and RAMP1 was more prominent, although, the CLR staining was weaker compared to that of RAMP1. The receptor components were detected in fibers within laminae I and II (Figure 12A,B). Double-staining of CLR and RAMP1 showed co-localization (Figure 12C). These fibers were different from fibers containing CGRP, revealed by double-staining of CGRP and the receptor components. Confocal microscopy disclosed that CGRP and the receptor components were not expressed in the same fibers (Figure 12D, additional file 2, movie). The receptor components, especially RAMP1, were also detected close to the central canal (laminae X) (Figure 12E). As for the STN of rat, the receptor components were found in capillary walls within the spinal cord at C1-level (Figure 12).

#### Controls and DAB staining

The negative controls (omission of primary antibody) showed no immunoreactivity, except for the lipofuscin autofluorescence in human samples (Figure 5, 7).

There was no immunoreactivity observed with preabsorbed CLR or RAMP1 antibodies, using their respective blocking peptides (additional file 3).

DAB stained sections showed similar staining pattern for CGRP, CLR and RAMP1 as seen with the immunofluorescence technique. DAB staining revealed CLR and RAMP1 expression in the walls of capillaries in rat (Figure 12E) and in laminae X, close to the central canal. Furthermore, three different secondary antibodies were tested to ensure that the capillary staining was not due to unspecific binding of the secondary antibody.

Two additional rat brainstems were kept at +4°C for 24 hrs before dissection, fixation and immunostaining to mimic the autopsy situation in man. As in our previous paper on the trigeminal ganglion [7]; this procedure did not differ in immunoreactivity for CGRP, CLR and RAMP1 as compared to fixation directly of fresh brainstems (data not shown).

## Discussion

We have previously reported in detail the distribution of CGRP and its receptor components in the human trigeminal ganglion [7]. Small to medium sized neurons of the trigeminal ganglion store CGRP and have further connections with STN in the brainstem, and in related extensions down to the C_1-2 _level via un-myelinated C-fibers [18]. CLR and RAMP1 are expressed in large neurons and thick fibers, which are co-expressed with a marker for Aδ-fibers. These fibers from the trigeminal ganglion extend further to the brainstem. Several studies point to the involvement of the brainstem in migraine pathophysiology [1,19] and a region within the brainstem has been demonstrated to be active during attacks in migraine patients [14,20].

The present study has revealed the detailed distribution and relations between CGRP and its receptor components in human and rat projection regions from the trigeminal ganglion. The study demonstrates for the first time the expression of CLR and RAMP1 within human STN in the brainstem and in the spinal cord at C1-level, using a series of novel well characterized antibodies. Thus, this indicates CGRP signaling in areas of the brainstem/spinal cord putatively involved in the migraine pathophysiology. The reason for choosing these regions in the present study is based on a series of detailed neuroanatomical mappings in rat showing pain pathways that may be involved in primary headaches [21].

### Distribution of CGRP, CLR and RAMP1 in STN

We observed CGRP expression around nerve fiber bundles in the superficial laminae of human and rat STN. We found no CGRP expressing neurons in the STN neither in man nor in rat. However, in rat we observed a small group of CGRP positive neurons located close to the central canal, in the inferior olive and in the hypoglossal nucleus of the brainstem. Notably these neurons were absent in man. The results were confirmed using 2 different antibodies against CGRP. The functional role of CGRP in these regions remains to be disclosed. The presence of CGRP in the brainstem is supported by early autoradiography studies [22,23]. In addition, CGRP expression in STN and spinal cord of different species has been studied [6,24-26]

The expression of CGRP in the brainstem differs between species [6]. It has been shown that the distribution of CGRP fibers is similar in rat and alpaca brainstem. However, CGRP containing neurons are more widespread in rat than in alpaca. In addition, the localization of CGRP positive neurons in the cat and alpaca brainstem differs [27]. Hence, the distribution pattern observed between rat and man in our study is likely due to species differences.

CGRP and MBP double-staining showed no co-localization, indicating that CGRP is as expected expressed in un-myelinated fibers in STN and this is in agreement with a previous study on the trigeminal ganglion [7]. Synaptophysin, used as a marker for synaptic vesicles, and CGRP were expressed in the same structures within the STN. This supports that CGRP is stored and released from nerve terminals as supported by a study on rat dorsal horn [28] and acts post-synaptic at CLR/RAMP1 expressing fibers.

In human STN, we found CLR and RAMP1 positive fibers mostly in the spinal trigeminal tract, spanning towards the superficial laminae. In rat STN, expression of CLR and RAMP1 were also found mostly in the spinal trigeminal tract, around fiber bundles and fibers in the spinal trigeminal tract spanning towards the superficial laminae. These results are in contrast to an earlier study by Lennerz *et al*., 2008, where CLR and RAMP1 expression was detected in the superficial laminae, partially co-localizing with CGRP. The reason for this difference could reside in that different antibodies were used.

There were no neuronal cell bodies immunopositive for CLR and RAMP1 in the human or rat STN. CLR and RAMP1 were observed to be co-expressed in the fibers, suggesting the presence of functional CGRP receptor in both types of species. Interestingly, we found that the receptor components were co-expressed on the walls of capillaries in rat STN

In rat STN, it was suggested that CGRP and its receptor components are localized in terminals from primary afferents [29]. In contrast, CLR did not co-localize with neuropeptides of primary spinal afferents in the dorsal horn of rat [30]. In the present work, we observed no co-localization between the receptor components and CGRP positive fibers, neither in man nor in rat. These results suggest that CGRP and the receptor components appear in nerve terminals, where C-fiber released CGRP may act post-synaptic at CGRP receptors on second-order neurons or modify the responses of trigeminal Aδ-fibers.

### Distribution of CGRP, CLR and RAMP1 in C1

We examined the localization and expression of CGRP and its receptor components in the spinal cord at the C1-level, since the main part of the trigeminovascular projection occurs at this level [21]. CGRP positive fibers were found in laminae I and II. Similar staining pattern has been demonstrated previously in the chick, quail dorsal horn of the spinal cord [31] and in the cat [32]. In addition, we observed some CGRP positive neurons and fibers close to the central canal (laminae X). This finding is in agreement with a previous study in rat spinal cord with neurons being positive for CGRP [6].

To determine which fibers express CGRP, co-localization experiments were performed with CGRP and IB4. IB4 has previously been used as a marker for C-fibers [30]. In the present study we found that IB4 and CGRP could be expressed in the same laminae, but most often in different types of fibers. This observation was confirmed by confocal microscopy. The IB4 staining was more prominent in the deeper laminae of the spinal cord, which is in agreement with a study of rat spinal cord [30]. It has been shown that the degree of co-expression of CGRP and IB4 in neurons vary in the rat. More neurons expressing both markers are found in dorsal root ganglia compared to the trigeminal ganglion of rat [33]. Electron microscopy showed that IB4 and CGRP expressing axons were distinct, but both could be present in the same bundle of un-myelinated fibers [34].

The MBP marker showed that in some areas within laminae I and II myelinated fibers are absent. Co-staining experiments with CGRP showed that CGRP is expressed in areas that are absent of MBP, suggesting that CGRP is indeed expressed in un-myelinated fibers.

To further scrutinize the CGRP positive fibers, double-staining of CGRP and synaptophysin was performed. In laminae I and II, where CGRP staining was found, co-expression of CGRP and synaptophysin was observed. With confocal microscopy, we obtained a detailed three-dimensional view of the staining pattern. This clearly showed that CGRP and synaptophysin were detected in the same fibers.

Reportedly, CLR and RAMP1 are expressed in fibers within laminae I and II in the dorsal horn of rat spinal cord [30]. Similar observation but in different tissue was seen in our study; CLR and RAMP1 expressions were found within laminae I and II of human and rat C1. No CLR or RAMP1 positive neuronal cell bodies were observed at the C1-level. In rat, CGRP was expressed in the same laminae, but it did not co-localize with CLR or RAMP1. CLR and RAMP1 were co-expressed, suggesting expression of functional CGRP receptor in fibers within laminae I and II. The presence of receptor components in the spinal cord is supported by the mRNA expression of RAMP1 and RCP, detected with specific oligonucleotides for *in situ *hybridization [35].

CLR and RAMP1 were in addition detected in fibers close to the central canal. As described above, CGRP positive fibers were also detected in this area. Interestingly, tracing experiments in cat have revealed projections from the periaqueductal gray (PAG) region to the spinal cord. Horseradish peroxidase (HRP) injections into the PAG region resulted in labeled fibers close to the central canal, terminating in laminae X of C1 and C2-levels [36]. The same authors found labeled fibers in segments C4 to T8 adjoining the ependymal layer of the central canal and next to the basal membrane of the nearby capillaries. Histochemical studies in different species have revealed neurons, axons and terminals within laminae X containing neuropeptides such as substance P [37]. Thus, our results demonstrate fibers containing CGRP and its receptor components close to the central canal. The function of this is not known; one may speculate that these fibers can release CGRP directly into the cerebrospinal fluid or stimulate the ependymal cells of the central canal.

### Methodology and technical considerations

The hematoxylin-eosin stained material revealed well-preserved human tissue adequate for immunofluorescence technique, even though the tissue samples were collected 24 to 36 hrs after death. Due to the relatively high age of the subjects, lipofuscin is accumulated in the tissue, causing auto-fluorescence. We have earlier examined, in rat trigeminal ganglion, if storage of the animals for 24 hrs at +4°C prior fixation would affect the immunohistochemistry; which was not the case [7]. Similar results were obtained in the present study on the rat brainstem (data not shown). In rat we also performed a direct comparison of perfusion-fixed and immersion-fixed brainstems, and found no observable difference in antibody expression pattern.

The C1-level compared well in the Paxinos atlas for rat and man. The STN is a structure that is distributed for a considerable length in the brainstem and could therefore not be examined for its entire distribution. It is a limitation of the present study that we only studied a portion of the STN; the exact part is given in the method part.

In our previous study, antibodies against human and rat CLR and RAMP1 were generated, and the specificity of the antibodies was confirmed in HEK293 cells stably expressing the human CGRP receptor. The specificity of the raised antibodies was also confirmed by Western blotting [7]. The same antibodies were used in this study.

The staining of the receptor components was weaker and a bit more diffuse in the human tissue compared to rat. This could be due of several factors: differences in antibodies recognizing the epitopes, tissue condition, or low level of CLR and RAMP1 in human tissue. In rat STN and C1, the RAMP1 antibody displayed a stronger staining pattern compared to the CLR antibody. If this was due to differences in antibody recognizing the epitopes or more expression of RAMP1 in these areas could not be verified.

Within the rat brainstem, we found expression of RAMP1 and CLR in the capillary walls. This was blocked with the specific blocking peptides (epitopes used in the production of the respective antibodies). The staining of the capillaries was similar in the endogenously activity blocked DAB-stained sections.

## Conclusions

The neuropeptide CGRP is implicated in the pathophysiology of migraine and the CGRP receptor has long been regarded as a useful target for the development of novel antimigraine therapies. We have described in detail CGRP and its receptor components in the STN and C1 of man and rat using immunohistochemistry. Fibers expressing CGRP and its receptor components occur in STN and C1, however they were not co-expressed in the different areas and laminae. This suggests that CGRP released from C-fibers in the brainstem may act postjunctionally to modulate the activity in fibers that store the CGRP receptor in these regions. Differences in the CGRP expression between the species were observed in other parts of the brainstem. We have also demonstrated fibers and neurons expressing CGRP close to the central canal which suggests that CGRP may have a function within this area. Further efforts are essential to understand CGRP signaling and its function within the brainstem.

## Abbreviations

BSA: Bovine serum albumin; CLR: Calcitonin receptor-like receptor; CGRP: Calcitonin gene-related peptide; DAB: 3,3'-diaminobenzidine; Htx-Eosin: Hematoxylin-Eosin; MBP: Myelin basic protein; NF: Nerve filament; PAG: Periaqueductal gray; PBS: Phosphate buffered-saline; PBST: Phosphate buffered-saline (PBS) containing 0.25% Triton X-100; PFA: Paraformaldehyde; RAMP1: Receptor activity-modifying protein 1; RCP: Receptor component protein; STN: Spinal trigeminal nucleus

## Authors' contributions

SE participated in the design of study, performed the experiments, analysed the data, prepared the figures and wrote the manuscript. LE conceived the study, guided the experimental procedures, and participated in writing the manuscript. Both authors read and approved the final manuscript.

## Supplementary Material

Additional file 1 **Movie: **3-dimensionally view of staining with CGRP (green) and IB4 (red) in rat C1. The two markers are most often not expressed in the same fibers. Occasionally the two markers co-localize in fibers (yellow). Click here for file

Additional file 2 **Movie: **3-dimensionally view of staining with CGRP (red) and RAMP1 (green) in rat C1. The neuropeptide CGRP and the receptor component RAMP1 are found within the same laminae, but they are not co-expressed in the same structures. Click here for file

Additional file 3 **Blocking peptide experiments**. CLR and RAMP1 antibodies were pre-absorbed with their respective blocking peptides. No positive immunoreactivity is found when the blocking peptides are used. Asterisks point at autofluorescent lipofuscin in the human samples. Human STN (A) preabsorption for CLR, (B) preabsorption for RAMP1. Rat STN (C) preabsorption for CLR, (D) preabsorption for RAMP1. Human C1 (E) preabsorption for CLR, (F) preabsorption for RAMP1. Rat C1 (G) preabsorption for CLR, (H) preabsorption for RAMP1. Click here for file
