# Supplementary figures and images for: Calcitonin gene-related peptide (CGRP) and its receptor components in human and rat spinal trigeminal nucleus and spinal cord at C1-level
Source: BMC Neurosci. 2011 Nov 10;12:112. doi: 10.1186/1471-2202-12-112 (PMC3282678; doi:10.1186/1471-2202-12-112)

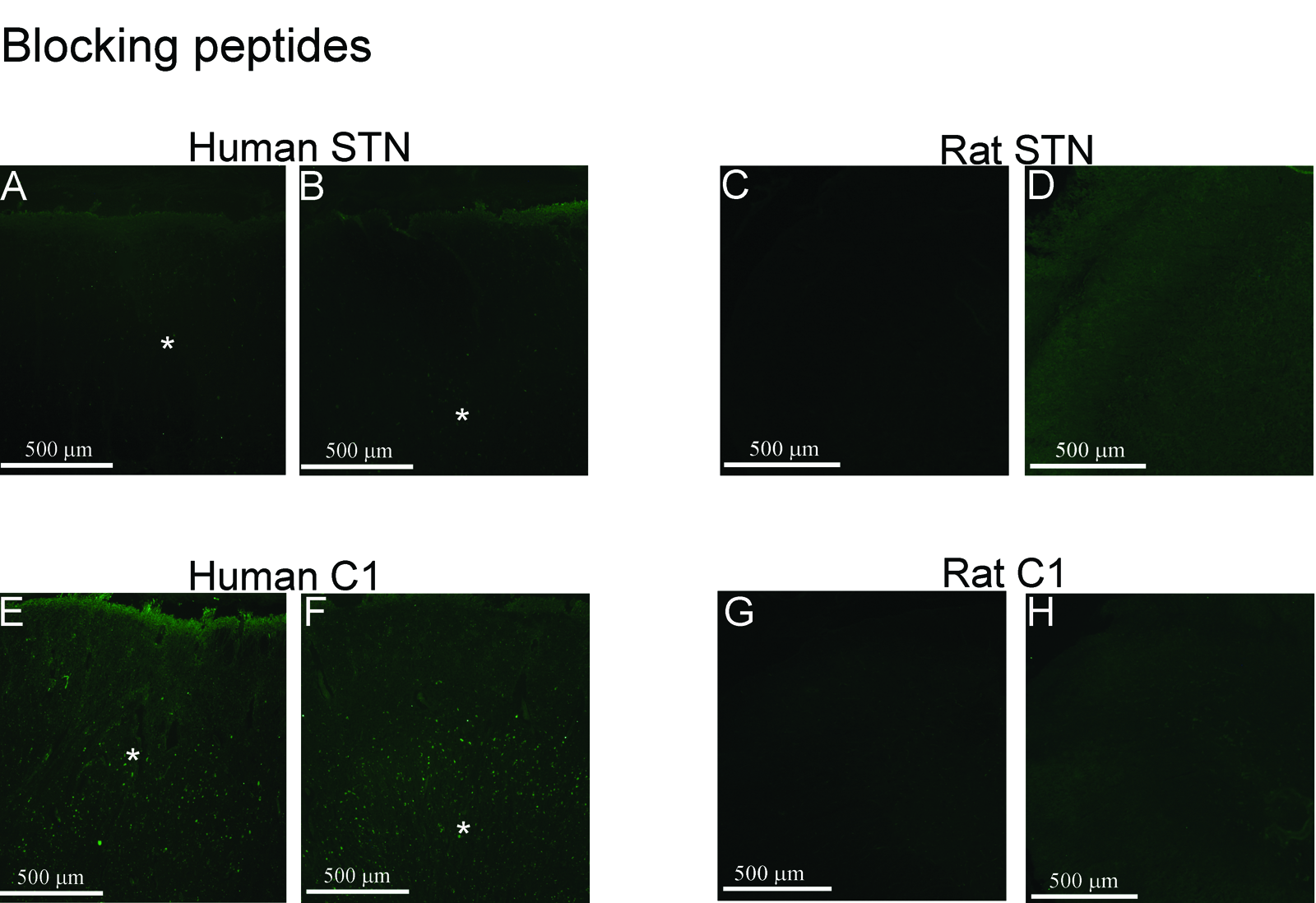

Supplement: Additional file 3 — Blocking peptide experiments. CLR and RAMP1 antibodies were pre-absorbed with their respective blocking peptides. No positive immunoreactivity is found when the blocking peptides are used. Asterisks point at autofluorescent lipofuscin in the human samples. Human STN (A) preabsorption for CLR, (B) preabsorption for RAMP1. Rat STN (C) preabsorption for CLR, (D) preabsorption for RAMP1. Human C1 (E) preabsorption for CLR, (F) preabsorption for RAMP1. Rat C1 (G) preabsorption for CLR, (H) preabsorption for RAMP1. [file 1471-2202-12-112-S3.TIFF]
